# Evaluation of the uptake and usability of the pregnant women surveillance system (SIMG): A cross‐sectional pilot study

**DOI:** 10.1002/ijgo.70996

**Published:** 2026-04-02

**Authors:** Charles M'poca Charles, Gabriel Valente Tozatti, Cristiano Torezzan, Renato Teixeira Souza, Maria Laura Costa, José Guilherme Cecatti, Rodolfo de Carvalho Pacagnella

**Affiliations:** ^1^ Department of Obstetrics and Gynecology Universidade Estadual de Campinas (UNICAMP) Campinas Brazil; ^2^ Provincial Health Administration, DPS Manica Chimoio Mozambique; ^3^ School of Applied Sciences (FCA) Universidade Estadual de Campinas (UNICAMP) Limeira Brazil

**Keywords:** COVID‐19 pandemic, pregnant women, risk assessment, surveillance system

In recent decades, technology, particularly the Internet, has transformed interactions and improved access to information, notably in women's health care.^1^ The COVID‐19 pandemic accelerated this shift, increasing demand for flexible care models and telehealth strategies.^2^, ^3^ However, there is a lack of knowledge regarding the barriers to the implementation, uptake, and usability of telehealth among pregnant women in the global south. Therefore, as part of the Brazilian Network for Studies on COVID‐19 in Obstetrics (REBRACO), we developed and piloted an Integrated Pregnancy Monitoring System (SIMG).^4^ This initiative aimed to enhance support for the integrated health care of pregnant women during the COVID‐19 pandemic through a telehealth tool. The objective of the pilot study was to assess the uptake and usability of the SIMG instrument. The SIMG is a web‐based application that uses expert systems for surveillance and clinical decision making. Accessible via mobile devices or browsers, it facilitates communication and data collection from patients. The tool employs decision trees and risk calculators to assist with risk stratification and identify potential obstetric complications during pregnancy and postpartum monitoring, flagging critical situations to both patients and healthcare providers. Participants in the REBRACO study used the SIMG for outpatient monitoring to assess COVID‐19 symptoms and evaluate severe morbidity risks in pregnant women (Figure S1).

At the time of enrollment in the REBRACO cohort, each participant was invited to register and received detailed instructions on how to use the SIMG remote follow‐up platform. Those who consented were able to self‐register directly within the tool, enabling longitudinal monitoring through remote self‐reporting. Between October and November 2022, we contacted 111 participants who had used the SIMG tool to evaluate the uptake and usability of the SIMG instrument. The study received ethical approval from the Institutional Review Board. After obtaining consent, participants completed the System Usability Scale (SUS) questionnaire online via Google Forms and participated in a telephone interview.^5^ The instrument can be used to assess various products and services, including websites, hardware, mobile applications, and medical systems.^6^

Out of 111 participants, 37 (33.3%) individuals did not respond to our invitation after three attempts, 14 (12.6%) chose not to participate in the assessment, and 6 (5.4 %) submitted incomplete information.

We conducted a descriptive analysis of sociodemographic, obstetric variables and the SIMG uptake (Table S1). The data from the SUS questionnaire were transformed into scores ranging from 0 to 100. Then, we performed a comparative analysis of the resulting SUS score, using a continuous score and three categories (acceptable, marginal, and unacceptable).^6^ We performed both univariate and multivariate linear regression analyses to examine the factors associated with SIMG uptake and usability. Uptake was defined as the proportion of invited participants who successfully registered and logged into the SIMG platform at least once during the study period.

A total of 54 (48.7%) participants were included in our analysis, with a mean age of 31 ± 6.9 years (mean ± standard deviation). Approximately 65% of the participants (35 out of 54) were aged between 18 and 35 years, resided in the southeastern region of Brazil, and had a partner (Table S1). The SIMG demonstrated marginal usability, with a mean score of 67.5 ± 19.8 (Figure 1a). Only 40.7% of participants (22 out of 54) scored above 70 on the SUS, indicating acceptable usability (Figure S2). Furthermore, the tool uptake was suboptimal (47.1%, 24 out of 51), and only 52.9% (27 out of 51) of participants used it more than once, indicating significant challenges regarding adherence (Figure 1b). However, almost 88% (47 out of 53) of respondents indicated a willingness to use the SIMG instrument in future pregnancies (Table S1). Additionally, the regression analysis indicated no significant differences in the SUS scores based on participant characteristics (Figures 1c,d and S3–S5).

**FIGURE 1:**
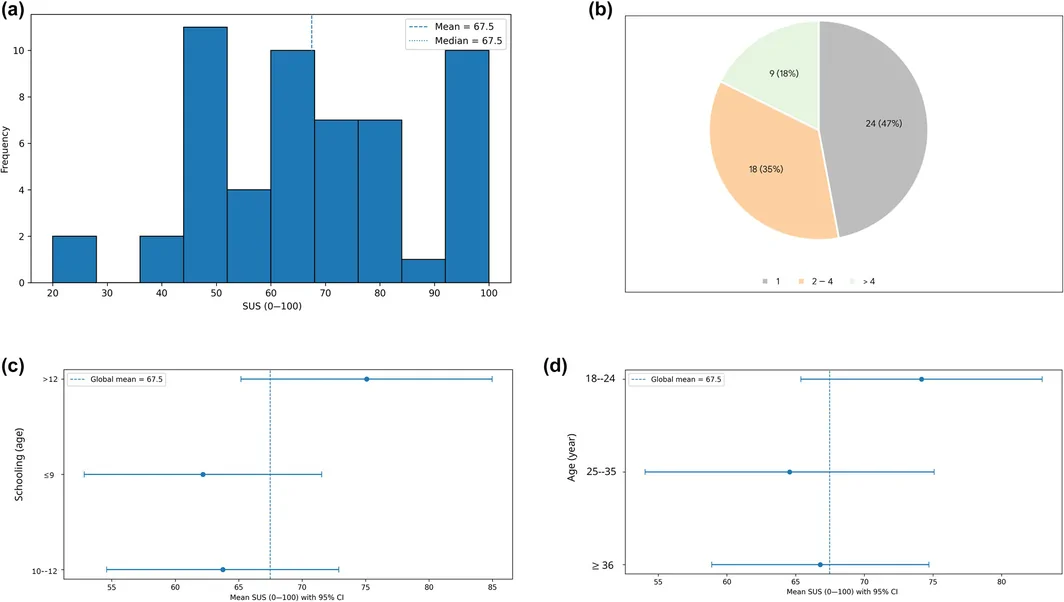
(a) Evaluation of the usability of the Integrated Pregnancy Monitoring System (SIMG) instrument and its factors among pregnant Brazilian women in referral hospitals. SUS, System Usability Scale, ranging from 0 to 100. (b) Frequency of SIMG instrument usage among pregnant women in referral hospitals in Brazil. (c) SUS score (mean ± 95% confidence intervals [CI]) among pregnant women using the SIMG instrument, categorized by schooling. (d) SUS score (mean ± 95% CI) among pregnant women using the SIMG instrument, categorized by age.

Several factors contributed to the low acceptance and utilization of the tool. Users often had limited time to engage consistently with the SIMG and faced challenges such as a lack of internet access and technologic illiteracy. Research has indicated that telehealth could exacerbate existing inequalities in maternal and perinatal health.^7^ Additionally, the implementation processes and procedures of the SIMG instrument may have significantly hindered user adoption. Our findings are supported by a Brazilian study that used The LifeAPP to monitor blood pressure in women after experiencing pre‐eclampsia. This study revealed that adherence to the app was below optimal levels.^8^ Additionally, existing literature indicate that while pregnant women value digital resources for information, their engagement often declines during active self‐monitoring tasks. Several factors may contribute to this decreased engagement, including technical issues, complicated protocols, and psychological barriers. These challenges highlight that moving beyond information‐seeking to active self‐monitoring requires user‐centered, holistic designs that prioritize seamless clinical integration over burdensome protocols.^9^, ^10^

The SIMG may have the potential to enhance prenatal care by facilitating the early detection of warning signs among expectant mothers and enabling healthcare services to extend their reach to a broader segment of the population without disrupting scheduled appointments. Furthermore, by fostering remote monitoring and communication, the program can mitigate delays in the identification of serious health conditions that contribute to elevated maternal and perinatal mortality rates. However, for this strategy to be successful, it is imperative to invest in the usability, engagement, and confidence required for the effective use of digital health tools.

## AUTHOR CONTRIBUTIONS

CT, CMC, and RCP collaborated in the development of the SIMG instrument. Each author made equal contributions to the writing and critical evaluation of the manuscript. They also conducted a thorough review and granted their approval of the final version.

## FUNDING INFORMATION

This study was financed, in part, by the São Paulo Research Foundation (FAPESP), Brazil. Process Number 2025/10862‐7, and the SRH, part of the UNDP‐UNFPA‐UNICEF‐WHO‐World Bank Special Programme for Research, Development, and Research Training in Human Reproduction, which is executed by the WHO. The study funders had no involvement in the study design, data collection, analysis, interpretation, report writing, or decision to submit the manuscript. Additionally, the study authors retained complete control of all primary data. This article solely reflects the views of the named authors and does not necessarily reflect those of the aforementioned organization.

## CONFLICT OF INTEREST STATEMENT

The authors have no conflicts of interest.

## Supporting information


**Figure S1.** Follow‐up periods for outpatient care of pregnant women using the SIMG instrument.
**Figure S2**. Evaluation of SIMG instrument usability by categorized System Usability Scale (SUS).
**Figure S3**. The SUS score (mean ± 95% CI) among pregnant women using the SIMG instrument, categorized by marital status.
**Figure S4**. The SUS score (mean ± 95% CI) among pregnant women using the SIMG instrument, categorized by family income.
**Figure S5**. The SUS score (mean ± 95% CI) among pregnant women using the SIMG instrument, categorized by regions.
**Table S1**. Sociodemographic characteristics of Brazilian pregnant women, SIMG usage frequency, and willingness to use it for future antenatal care by SUS score.

## Data Availability

The data that support the findings of this study are available on request from the corresponding author. The data are not publicly available due to privacy or ethical restrictions.
