# Supplementary material for: Evaluation of the uptake and usability of the pregnant women surveillance system (SIMG): A cross‐sectional pilot study
Source: Int J Gynaecol Obstet. 2026 Apr 2;175(1):481–3. doi: 10.1002/ijgo.70996 (PMC13629578; doi:10.1002/ijgo.70996)
Supplement: Supplementary file 1 [file IJGO-175-481-s001.docx]

**Evaluation of the uptake and usability of the Pregnant Women Surveillance System (SIMG): A Cross-Sectional Pilot Study.**

**Supplementary materials**

**SMF1:** Follow-up periods for outpatient care of pregnant women using the SIMG instrument.

**Legend:** Wks – weeks, PP – postpartum. The SIMG was established to connect pregnant patients with medical teams using information systems and artificial intelligence. This facilitates care through telemedicine. Participants were encouraged to access and use the system on a daily basis or as needed. Additionally, they were advised to attend regular antenatal care visits in accordance with local and context-specific protocols. The figure was created in https://BioRender.com.

**SMF2:** Evaluation of SIMG instrument usability by categorized System Usability Scale (SUS)

**SMF3:** The SUS score (mean±95% CI) among pregnant women using the SIMG instrument, categorized by marital status.

**SMF4:** The SUS score (mean±95% CI) among pregnant women using the SIMG instrument, categorized by family income**.**

**SMF5:** The SUS score (mean±95% CI) among pregnant women using the SIMG instrument, categorized by regions.

**SMT1:** Sociodemographic characteristics of Brazilian pregnant women, SIMG usage frequency, and willingness to use it for future antenatal care by SUS Score.

| **Characteristics** | **N** | **SUS Score categories** | | |
| --- | --- | --- | --- | --- |
|  |  | **Unacceptable**  N (%) | **Marginal**  **N (%)** | **Acceptable**  N (%) |
| **Age** (years) |  |  |  |  |
| 18-24 |  | 0 (0.0) | 6 (23.1) | 6 (27.3) |
| 25-35 |  | 6 (100.0) | 9 (34.6) | 8 (36.4) |
| ≥ 36 |  | 0 (0.0) | 11 (42.33) | 8 (36.4) |
| **Schooling (years)** |  |  |  |  |
| ≤ 9 |  | 3 (50.0) | 8 (30.8) | 5 (22.7) |
| 10 - 12 |  | 2 (33.3) | 12 (38.5) | 6 (27.3) |
| ˃ 12 |  | 1 (16.7) | 8 (30.8) | 11 (50.0) |
| **Marital status** |  |  |  |  |
| With partner |  | 5 (83.3) | 16 (61.5) | 15 (68.2) |
| Without partner |  | 1 (16.7) | 10 (38.5) | 7 (31.8) |
| **Occupation ^a)^** |  |  |  |  |
| Employed |  | 5 (83.3) | 17 (70.8) | 14 (73.7) |
| Unemployed |  | 0 (0.0) | 2 (8.3) | 2 (10.5) |
| Student |  | 1 (16.7) | 5 (20.8) | 3 (15.8) |
| **Family income (**USD**)** |  |  |  |  |
| ≤200 |  | 3 (50.0) | 11 (42.3) | 8 (36.4) |
| 201 - 565 |  | 3 (50.0) | 8 (30.8) | 7 (31.8) |
| 566 – 945 |  | 0 (0.0) | 2 (7.7) | 3 (13.6) |
| 946 – 1320 |  | 0 (0.0) | 5 (19.2) | 4 (18.2) |
| ˃ 1320 |  |  |  |  |
| **Region** |  |  |  |  |
| North, Northeast and Midwest |  | 1 (16.7) | 10 (38.5) | 8 (36.4) |
| South and Southeast |  | 5 (83.3) | 16 (61.5) | 14 (63.6) |
| **Frequency of use ^b)^** |  |  |  |  |
| 1 |  | 11 (57.9) | 7 (63.6) | 6 (28.6) |
| 2 - 4 |  | 2 (10.5) | 4 (36.4) | 12 (57.1) |
| ˃ 4 |  | 6 (31.6) | 0 (0.0) | 3 (14.3) |
| **Use of SIMG for ANC follow-up ^c)^** |  |  |  |  |
| No |  | 1 (5.3) | 0 (0.0) | 2 (9.1) |
| Maybe |  | 1 (5.3) | 0 (0.0) | 2 (9.1) |
| Yes |  | 17 (89.5) | 12 (100) | 18 (81.8) |

Missing value: a) 5, b) 3, c) 1
